# Energy Intake, Profile, and Dietary Sources in the Spanish Population: Findings of the ANIBES Study

**DOI:** 10.3390/nu7064739

**Published:** 2015-06-12

**Authors:** Emma Ruiz, José Manuel Ávila, Teresa Valero, Susana del Pozo, Paula Rodriguez, Javier Aranceta-Bartrina, Ángel Gil, Marcela González-Gross, Rosa M. Ortega, Lluis Serra-Majem, Gregorio Varela-Moreiras

**Affiliations:** 1 Spanish Nutrition Foundation (FEN), C/General Álvarez de Castro 20, 1ªpta, 28010 Madrid, Spain; E-Mails: eruiz@fen.org.es (E.R.); jmavila@fen.org.es (J.M.Á.); tvalero@fen.org.es (T.V.); susanadelpozo@fen.org.es (S.P.); prodriguez@fen.org.es (P.R.); 2 Department of Preventive Medicine and Public Health, University of Navarra, C/Irunlarrea 1, 31008 Pamplona, Spain; E-Mail: jaranceta@unav.es; 3 Department of Biochemistry and Molecular Biology II and Institute of Nutrition and Food Sciences, University of Granada, Campus de la Salud, Avda. del Conocimiento, 18100 Armilla, Granada, Spain; E-Mail: agil@ugr.es; 4 ImFINE Research Group, Department of Health and Human Performance, Technical University of Madrid, C/Martín Fierro 7, 28040 Madrid, Spain; E-Mail: marcela.gonzalez.gross@upm.es; 5 Department of Nutrition, Faculty of Pharmacy, Complutense University of Madrid, Plaza Ramón y Cajal s/n, 28040 Madrid, Spain; E-Mail: rortega@ucm.es; 6 Research Institute of Biomedical and Health Sciences, Universidad de Las Palmas de Gran Canaria, Facultad de Ciencias de la Salud, C/Doctor Pasteur s/n Trasera del Hospital, 35016 Las Palmas de Gran Canaria, Spain; E-Mail: lluis.serra@ulpgc.es; 7 Department of Pharmaceutical and Health Sciences, Faculty of Pharmacy, CEU San Pablo University, Urb. Montepríncipe, Crta. Boadilla Km. 5.3, 28668 Boadilla del Monte, Madrid, Spain

**Keywords:** energy intake, dietary energy sources, dietary surveys, food intake, ANIBES study

## Abstract

Energy intake, and the foods and beverages contributing to that, are considered key to understanding the high obesity prevalence worldwide. The relative contributions of energy intake and expenditure to the obesity epidemic, however, remain poorly defined in Spain. The purpose of this study was to contribute to updating data of dietary energy intake and its main sources from food and beverages, according to gender and age. These data were derived from the ANIBES (“Anthropometry, Intake, and Energy Balance in Spain”) study, a cross-sectional study of a nationally representative sample of the Spanish population (from 9–75 years old). A three-day dietary record, collected by means of a tablet device, was used to obtain information about food and beverage consumption and leftovers. The final sample comprised 2009 individuals (1,013 men, 996 women). The observed mean dietary energy intake was 7.6 ± 2.11 MJ/day (8.2 ± 2.22 MJ/day for men and 6.9 ± 1.79 MJ/day for women). The highest intakes were observed among adolescents aged 13–17 years (8.4 MJ/day), followed by children 9–12 years (8.2 ± 1.80 MJ/day), adults aged 18–64 (7.6 ± 2.14 MJ/day) and older adults aged 65–75 years (6.8 ± 1.88 MJ/day). Cereals or grains (27.4%), meats and derivatives (15.2%), oils and fats (12.3%), and milk and dairy products (11.8%) contributed most to daily energy intake. Energy contributions from non-alcoholic beverages (3.9%), fish and shellfish (3.6%), sugars and sweets (3.3%) and alcoholic beverages (2.6%) were moderate to minor. Contributions to caloric profile were 16.8%E from proteins; 41.1%E from carbohydrates, including 1.4%E from fiber; 38.5%E from fats; and 1.9%E from alcohol intake. We can conclude that energy intake is decreasing in the Spanish population. A variety of food and beverage groups contribute to energy intake; however, it is necessary to reinforce efforts for better adherence to the traditional Mediterranean diet.

## 1. Introduction

In Europe, national and regional lifestyle practices, including dietary habits, have been changing over the past 50 years, becoming less distinct and moving towards a more homogeneous “Western diet” [1,2,3]. Spain has undergone dramatic social and socioeconomic change since the 1960s, including massive rural–urban migration, rapid urbanization processes during the 1980s, and generalized incorporation of females into the active workforce [4]. As a result of these transitions, the Spanish population has partially turned away from its traditional Mediterranean diet. The changes in diet, physical activity, and lifestyle seem to have had potentially negative consequences for both present and future populations. Overweight and/or obesity affect more than 50% of the adult population and nearly 30% of the population of infants and children [5]. It has been suggested that excessive energy intake is the primary cause of overweight and obesity. However, a sedentary lifestyle and lack of physical activity are thought to have at least as important a role as diet in the etiology of obesity [4,6].

The methodologies and procedures used in dietary surveys have been widely developed with the aim of evaluating the nutritional status of a population [7,8]. Problems associated with such studies are difficulties in terms of methodology, relative participation (high level of respondent commitment, biased sampling, and others), collecting intake data (truthfulness, forgetfulness, and others) and quantifying amounts consumed (portion size, ingredients in recipes, and others) [9]. Therefore, using new available methodologies (e.g., “real-time” recording of eating/drinking events) to avoid the usual bias is challenging, but urgently needed [10,11,12]. Moreover, there is consensus that determinants of diet, food composition, and consumption remain largely unknown*,* a fact that has become more true as related factors have become more complex, such as origin, production, availability, gastronomic trends, and others [7,12]. In this regard, there is a need to improve tools such as food composition tables and databases that include detailed information on composition of the different food and beverage groups and subgroups representative of the current Spanish food basket, as well as to update energy requirements and serving sizes. We first stated this need in 2013, in the consensus document and conclusions on “Obesity and Sedentarism in the 21st Century: what can be done and what must be done?” [4] and, more recently, in the “Consensus Meeting on the Methodology of Dietary Surveys, Classification of Physical Activity and Healthy Lifestyles” [13].

Many valuable dietary surveys have been previously conducted in Spain [14,15,16,17,18,19,20], although to the best of our knowledge, no one has approached energy intake and its determinants using new, more accurate technologies. To fill this gap, the ANIBES (“Anthropometry, Intake, and Energy Balance in Spain”) study was specifically designed to focus on energy balance and its determinants in Spain. The main objective of the present study was to analyze energy intake in a nationally representative sample of the Spanish population, its distribution by sex and age groups, and to identify those food and beverages sources that contribute to energy intake.

## 2. Materials and Methods

The design, protocol, and methodology of the ANIBES study have been already described in detail elsewhere [21,22].

### 2.1. Sample

The ANIBES study was conducted using stratified multistage sampling. To guarantee better coverage and representativeness, the fieldwork was performed at 128 sampling points all across Spain. No pre-recruitment was considered so as to minimize the risk of bias in responses. The design of the ANIBES study aimed to define a sample size that is representative of all individuals living in Spain, aged 9 to 75 years, and living in municipalities of at least 2000 inhabitants. The initial potential sample consisted of 2634 individuals, and the final sample comprised 2009 individuals (1013 men, 50.4%; 996 women, 49.6%). In addition, for the youngest age groups (9–12, 13–17, and 18–24 years), a boost sample was included to have at least *n =* 200 per age group (error +/−6.9%). Therefore, the random sample plus booster comprised 2285 participants.

The sample quotas according to the following variables were: age groups (9–12, 13–17, 18–64, and 65–75 years); sex (men/women); geographical distribution (Northeast, Levant, Southwest, North-Central, Barcelona, Madrid, Balearic and Canary Islands); and locality size: 2000 to 30,000 inhabitants (rural population); 30,000 to 200,000 inhabitants (semi-urban population) and over 200,000 inhabitants (urban population). Additionally, other factors for sample adjustment were considered: unemployment rate, percentage of foreigners (immigrant population), physical activity level, and education or economic level.

The fieldwork for the ANIBES study was conducted from mid-September 2013 to mid-November 2013, and two previous pilot studies were also performed. To equally represent all days of the week, subjects participated during two weekdays and one weekend day. The final protocol was approved by the Ethical Committee for Clinical Research of the Region of Madrid (Spain).

### 2.2. Food and Beverage Record

Study participants were provided with a tablet device (Samsung Galaxy Tab 2 7.0) and trained in how to record information by taking photos of all food and drinks consumed during the three days of the study, both at home and outside the home. Photos had to be taken before beginning to eat and drink, and again after finishing, so as to record the actual intake. Additionally, a brief description of meals, recipes, brands, and other data was recorded using the device. Participants who declared or demonstrated that they were unable to use the tablet device were offered other options, such as using a digital camera and paper record and/or conducting telephone interviews. A total 79% of the sample used a tablet, 12% a digital camera, and 9% opted for a telephone interview. In addition to details of what and how much was eaten, for each eating/drinking event participants recorded where they were, who they were eating with, and whether they were watching television and/or sitting at a table. After each survey day, participants recorded if their intake was representative for that day (or the reason why if it was not), and details of any dietary supplements taken. The survey also contained a series of questions about participants’ customary eating habits (e.g., the type of milk or fat spread usually consumed) to facilitate further coding. Food records were returned from the field in real time, to be coded by trained coders who were supervised by dieticians. An ad hoc central server software/database was developed for this purpose, to be able to work in parallel with the codification and verification processes. The software was developed to receive information from the field tablets every 2s, and the database was updated every 30 min. Food, beverages, energy and nutrient intakes were calculated from food consumption records using this software (VD-FEN 2.1), which was newly developed for the ANIBES study by the Spanish Nutrition Foundation and is based mainly on Spanish food composition tables [23], with several expansions and updates. Data obtained from food manufacturers and nutritional information provided on food labels were also included. A food photographic atlas was used to assist in assigning gram weights to portion sizes. Energy distribution objectives for the Spanish population were used to analyze the overall quality of the diet [24].

### 2.3. Statistical Analysis 

Once all dietary intake information was transformed into energy and nutrient data, these data were processed using different statistical analysis tools and packages. The following statistics were calculated to qualify each variable in the analysis: average, standard deviation, and variance to measure dispersion in the values; minimum and maximum values, median, quartiles (as well as interquartile range), and deciles to describe the shape of the distribution. The Kolmogorov–Smirnoff test was used to test normality of the distribution. In addition, the intake data were grouped into 14 food groups, 45 subgroups and 754 ingredients for in-depth analysis.

**Table 1:** Total daily energy intake, by sex and age group, in the ANIBES survey of the Spanish population aged 9–75 years, expressed as kilocalories and megajoules.

ENERGY (kcal/day)	Total *				Children 9–12 Years *				Adolescents 13–17 Years *				Adults 18–64 Years *				Elderly 65–75 Years *			
	*n*	Mean	SD	SEM	*n*	Mean	SD	SEM	*n*	Mean	SD	SEM	*n*	Mean	SD	SEM	*n*	Mean	SD	SEM
**Total**	2009	**1810**	*504.4*	11.25	213	**1960**	*431.3*	29.6	211	**2018**	*508.1*	35.0	1655	**1816**	*512.0*	12.6	206	**1618**	*448.4*	31.2
Men	1013	**1957**	*531.0*	16.68	126	**2006**	*456.1*	40.6	137	2124	*514.6*	44.0	798	**1966**	*543.2*	19.2	99	**1771**	*484.7*	48.7
Women	996	**1660**	*426.7*	13.52	87	**1893**	*385.5*	41.3	74	**1823**	*435.7*	50.6	857	**1675**	*436.9*	14.9	107	**1476**	*359.9*	34.8
**ENERGY (MJ/day)**	Tota l *				Children 9–12 Years *				Adolescents 13–17 Years *				Adults 18–64 Years *				Elderly 65–75 Years *			
	***n***	**Mean**	**SD**	**SEM**	***n***	**Mean**	**SD**	**SEM**	***n***	**Mean**	**SD**	**SEM**	***n***	**Mean**	**SD**	**SEM**	***n***	**Mean**	**SD**	**SEM**
**Total**	2009	**7.6**	*2.11*	0.05	213	**8.2**	*1.80*	0.12	211	**8.4**	*2.13*	0.15	1655	**7.6**	*2.14*	0.05	206	**6.8**	*1.88*	0.13
Men	1013	**8.2**	*2.22*	0.07	126	**8.4**	*1.91*	0.17	137	**8.9**	*2.15*	0.18	798	**8.2**	*2.27*	0.08	99	**7.4**	*2.03*	0.20
Women	996	**6.9**	*1.79*	0.06	87	**7.9**	*1.61*	0.17	74	**7.6**	*1.82*	0.21	857	**7.0**	*1.83*	0.06	107	**6.2**	*1.51*	0.15

* Denotes statistical difference (*p* ≤ 0.05) by sex; SD: mean standard deviation; SEM: mean standard error.

**Table 2:** Energy distribution (kcal/day; MJ/day), by age group and sex, in the ANIBES survey of the Spanish population aged 9–75 years.

Energy (kcal/day)	*n*	Mean	Median	SD	SEM	P5	P10	P25	P50	P75	P90	P95	Minimum	Maximum
Total *	**2009**	**1810**	**1756**	***504.4***	**11.3**	**1092**	**1217**	**1440**	**1756**	**2109**	**2487**	**2717**	**799**	**4819**
Men	1013	1957	1907	*531.0*	16.7	1190	1320	1558	1907	2296	2641	2926	799	4819
Women	996	1660	1620	*426.7*	13.5	1030	1146	1337	1620	1930	2214	2415	801	3340
Children 9–12 years *	**213**	**1960**	**1954**	***431.3***	**29.6**	**1301**	**1428**	**1651**	**1954**	**2189**	**2565**	**2712**	**538**	**3519**
Men	126	2006	1987	*456.1*	40.6	1232	1481	1759	1987	2307	2625	2733	538	3519
Women	87	1893	1937	*385.5*	41.3	1338	1404	1608	1937	2117	2349	2526	1021	3048
Adolescents 13–17 years *	**211**	**2018**	**1972**	***508.1***	**35.0**	**1266**	**1412**	**1624**	**1972**	**2334**	**2683**	**2909**	**799**	**3572**
Men	137	2124	2140	*514.6*	44.0	1410	1480	1752	2140	2459	2759	2952	799	3572
Women	74	1823	1819	*435.7*	50.6	1176	1299	1525	1819	2057	2253	2633	1049	3127
Adults 18–64 years *	**1655**	**1816**	**1758**	***512.0***	**12.6**	**1091**	**1214**	**1435**	**1758**	**2118**	**2501**	**2742**	**801**	**4819**
Men	798	1966	1919	*543.2*	19.2	1184	1309	1557	1919	2326	2649	2954	801	4819
Women	857	1675	1648	*436.9*	14.9	1030	1141	1340	1648	1936	2259	2454	801	3340
Elderly 65–75 years *	**206**	**1618**	**1531**	***448.4***	**31.2**	**1016**	**1122**	**1308**	**1531**	**1882**	**2147**	**2278**	**874**	**3899**
Men	99	1771	1711	*484.7*	48.7	1101	1256	1481	1711	2003	2253	2697	874	3899
Women	107	1476	1426	*359.9*	34.8	965	1065	1247	1426	1627	1996	2133	886	2800

* Denotes statistical difference (*p* ≤ 0.05) by sex; SD: mean standard deviation; SEM: mean standard error.

**Table 3:** Energy and lipid profile (%) in the ANIBES survey of the Spanish population aged 9–75 years, by sex and age group.

	Total			Children			Adolescents			Adults			Elderly		
	9–75 Years			9–12 Years			13–17 Years			18–64 Years			65–75 Years		
	Total	Men	Women	Total	Men	Women	Total	Men	Women	Total	Men	Women	Total	Men	Women
***n***	**2009**	1013	996	**213**	126	87	**211**	137	74	**1655**	798	857	**206**	99	107
**Mean energy intake (kcal/day)**	**1810**	**1957**	**1660**	**1960**	**2006**	**1893**	**2018**	**2124**	**1823**	**1816**	**1966**	**1675**	**1618**	**1771**	**1476**
**(%) Proteins**	16.8 *	16.7	17.0	**16.0**	16.3	15.6	**16.2**	16.4	15.9	**16.9**	16.8	17.0	**17.1**	16.9	17.3
**(%) Carbohydrates**	**41.1**	41.0	41.2	**43.8**	43.4	44.4	**44.4**	43.9	45.2	**40.7**	40.6	40.9	40.7 *	39.6	41.7
(%) Sugars	17.0 *	16.3	17.8	**18.8**	18.8	18.8	17.7 *	16.9	19.2	16.7 *	16.0	17.3	18.3 *	16.7	19.8
**(%) Lipids**	**38.5**	38.2	38.7	**38.9**	39.0	38.6	**38.1**	38.4	37.5	38.6 *	38.2	39.0	**37.2**	37.0	37.4
(%) SFA	**11.7**	11.6	11.7	**13.1**	13.2	12.9	**12.5**	12.6	12.2	**11.7**	11.5	11.8	**10.6**	10.5	10.7
(%) MUFA	**16.8**	16.6	16.9	**16.0**	16.1	15.8	**15.7**	15.9	15.4	16.8 *	16.6	17.0	**17.1**	17.0	17.2
(%) PUFA	**6.63**	6.6	6.6	**6.4**	6.3	6.5	**6.4**	6.4	6.5	**6.7**	6.6	6.7	**6.2**	6.2	6.1
(%) *n*-6	**5.40**	5.43	5.37	**5.44**	5.36	5.55	**5.53**	5.53	5.54	**5.45**	5.48	5.43	**4.90**	4.87	4.92
(%) *n*-3	**0.63**	0.72	0.55	**0.44**	0.43	0.45	**0.45**	0.45	0.46	**0.66**	0.77	0.55	**0.62**	0.66	0.57
(%) Alcohol	1.9 *	2.5	1.4	**0.0**	0.0	0.0	**0.0**	0.0	0.1	2.1 *	2.8	1.5	2.7 *	4.1	1.4
(%) Fiber	1.4 *	1.4	1.5	1.2 *	1.2	1.3	**1.2**	1.2	1.2	1.4 *	1.4	1.5	**1.8**	1.8	1.9

* Denotes statistical difference (*P* ≤ 0.05) by sex; SD: mean standard deviation; SEM: mean standard error.

## 3. Results

### 3.1. Total Energy Intake, Profile, and Distribution

Mean daily energy intakes for total energy for the entire Spanish population aged 9–75 years are shown in Table 1. Males had statistically higher intakes than females (*p <* 0.05) for the whole sample. By age group, adolescents and elderly males had higher intakes than females (*p <* 0.05).

Table 2 shows the energy intake distribution (median, percentiles, and maximum/minimum) according to age group and sex. In terms of the contribution of macronutrients to dietary energy (Table 3), carbohydrates contributed the highest proportion (41.1%), followed by fats (38.5%) and proteins (16.8%); other minor energy sources were alcohol (1.9%) and fiber (1.4%). Women had higher sugar and fat intakes (*p* < 0.05) and men higher alcohol intakes (*p* < 0.05). Concerning the energy provided by fatty acids, monounsaturated fatty acids (MUFA) contributed 16.8%, saturated fatty acids (SFA) 11.7%, and polyunsaturated fatty acids (PUFA) 6.6% (5.40% *n*-6 class; 0.63% *n*-3). No gender differences were observed for the lipid profile. However, there were differences according to age group: SFA contribution to energy intake was highest for children (13.1%) and lowest for the oldest age group (10.6%). The opposite was seen for MUFA, with the highest contribution for elderly adults. There were no differences for PUFA between age groups (Table 3).

### 3.2. Contribution of Food and Beverage Groups to Total Intake

The contribution (%) of food and beverage categories to the daily energy intake is shown in Table 4, ranked from high to low, and categorized by age group.

**Table 4:** Dietary sources of energy (%) from food groups/subgroups in the ANIBES survey of the Spanish population aged 9–75 years.

% Energy	Total 9–75	Children 9–12	Adolescents 13–17	Adults 18–64	Elderly 65–75
***n***	**2009**	**213**	**211**	**1655**	**206**
**Energy (kcal/day)**	**1810**	**1960**	**2018**	**1816**	**1618**
**Cereals/Grains**	27.4	30.4	31.1	27.9	26.2
*Bread*	11.6	11.3	11.5	11.8	12.5
*Bakery and pastry*	6.8	9.4	8.4	6.8	6.4
*Grains and flours*	4.5	4.0	4.7	4.6	4.1
*Pasta*	3.6	4.1	4.5	3.7	2.5
*Breakfast cereals and cereal bars*	1.0	1.6	2.0	1.0	0.8
**Meat and meat products**	15.2	15.3	16.2	15.7	13.1
*Meat*	9.2	8.2	9.2	9.6	8.5
*Sausages and other meat products*	5.8	7.1	7.0	5.9	4.5
*Viscera and offal*	0.1	0.0	0.0	0.1	0.2
**Oils and fats**	12.3	10.4	9.8	12.2	14.9
Olive oil	9.2	7.1	6.6	9.1	12.2
Other oils	1.7	2.0	1.9	1.8	0.8
Butter, margarine and shortening	1.4	1.3	1.2	1.3	1.8
**Milk and dairy products**	11.8	15.9	12.8	11.8	12.2
*Milk*	5.0	6.9	5.9	4.9	5.9
*Cheese*	3.0	2.6	2.9	3.2	2.3
*Yogurt and fermented milk*	2.4	3.1	2.0	2.3	3.1
*Other dairy products*	1.5	3.2	2.0	1.5	0.8
**Fruits**	4.7	3.0	2.4	4.7	8.7
**Ready-to-eat-meals**	4.2	5.7	6.6	4.3	1.8
**Vegetables**	4.0	3.0	3.0	4.2	5.0
**Non-alcoholic beverages**	3.9	4.9	6.1	3.9	2.2
*Sugared soft drinks*	2.0	1.9	3.4	2.1	0.7
*Juices and nectars*	1.3	2.9	2.5	1.3	0.9
*Other drinks*	0.3	0.0	0.1	0.3	0.3
*Coffee and herbal teas*	0.2	0.0	0.0	0.2	0.3
*Sports drinks*	0.1	0.2	0.0	0.1	0.0
*Energy drinks*	0.0	0.0	0.1	0.0	0.0
*Unsweetened soft drinks*	0.0	0.0	0.0	0.0	0.0
**Fish and shellfish**	3.6	2.2	2.1	3.7	4.7
**Sugars and sweets**	3.3	5.1	4.4	3.3	2.6
*Chocolate*	1.5	4.2	3.3	1.4	0.5
*Sugar*	1.4	0.5	0.7	1.5	1.4
*Jams and other*	0.3	0.2	0.1	0.3	0.8
*Other sweets*	0.1	0.2	0.2	0.1	0.0
**Alcoholic beverages**	2.6	0.0	0.0	2.9	3.5
Low alcohol content beverages	2.4	0.0	0.0	2.6	3.3
High alcohol content beverages	0.2	0.0	0.0	0.3	0.3
**Pulses**	2.2	2.0	2.0	2.3	2.9
**Eggs**	2.2	2.0	2.1	2.2	2.8
**Sauces and condiments**	1.6	1.4	1.7	1.7	0.9
**Appetizers**	0.8	1.1	1.0	0.8	0.3
**Supplements and meal replacements**	0.1	0.0	0.0	0.1	0.1

A detailed analysis of the food and beverage groups and subgroups, by sex, is shown in Table 5, Table 6, Table 7, Table 8 and Table 9.

**Table 5:** Dietary sources of energy (%) from food subgroups in the ANIBES survey of the Spanish population aged 9–75 years, by sex.

	Total 9–75 Years		
	Total	Men	Women
*n*	2009	1013	996
Energy (kcal/day)	1810	1957	1660
Bread	11.6	12.2	11.0
Meat	9.2	9.7	8.8
Olive oil	9.2	8.7	9.8
Bakery and pastry	6.8	6.5	7.1
Sausages and other meat products	5.8	6.3	5.2
Milk	5.0	4.6	5.3
Fruits	4.7	4.2	5.3
Grains and flours	4.5	4.4	4.5
Ready-to-eat-meals	4.2	4.6	3.8
Vegetables	4.0	3.8	4.3
Pasta	3.6	3.7	3.4
Fish and shellfish	3.6	3.2	3.9
Cheese	3.0	2.9	3.1
Low alcohol content beverages	2.4	3.1	1.7
Yogurt and fermented milk	2.4	2.2	2.6
Pulses	2.2	2.2	2.3
Eggs	2.2	2.3	2.1
Sugared soft drinks	2.0	2.2	1.8
Other oils	1.7	1.7	1.7
Sauces and condiments	1.6	1.6	1.5
Chocolate	1.5	1.6	1.4
Other dairy products	1.5	1.5	1.6
Sugar	1.4	1.2	1.5
Butter, margarine and shortening	1.4	1.1	1.6
Juices and nectars	1.3	1.4	1.3
Breakfast cereals and cereal bars	1.0	1.0	1.1
Appetizers	0.8	0.8	0.8
Jams and other	0.3	0.2	0.4
Other drinks (non-alcoholic)	0.3	0.2	0.3
High alcohol content beverages	0.2	0.3	0.2
Coffee and herbal teas	0.2	0.2	0.2
Viscera and offal	0.1	0.1	0.1
Other sweets	0.1	0.1	0.1
Supplements and meal replacements	0.1	0.1	0.1
Sports drinks	0.1	0.1	0.0
Energy drinks	0.0	0.1	0.0
Unsweetened soft drinks	0.0	0.0	0.0

**Table 6:** Dietary sources of energy (%) from food subgroups in children from the ANIBES survey of the Spanish population.

	Children 9–12 Years		
	Total	Men	Women
*n*	213	126	87
Energy (kcal/day)	1960	2006	1893
Bread	11.3	11.2	11.4
Bakery and pastry	9.4	9.1	10.0
Meat	8.2	8.9	7.2
Sausages and other meat products	7.1	7.1	7.1
Olive oil	7.1	7.0	7.2
Milk	6.9	7.3	6.3
Ready-to-eat-meals	5.7	6.0	5.2
Chocolate	4.2	4.1	4.3
Pasta	4.1	3.9	4.3
Grains and flours	4.0	3.7	4.4
Other dairy products	3.2	3.4	2.9
Yogurt and fermented milk	3.1	3.1	3.2
Vegetables	3.0	2.9	3.2
Fruits	3.0	2.7	3.4
Juices and nectars	2.9	2.8	3.1
Cheese	2.6	2.6	2.6
Fish and shellfish	2.2	2.0	2.5
Pulses	2.0	1.7	2.5
Other oils	2.0	1.9	2.1
Eggs	2.0	2.0	1.9
Sugared soft drinks	1.9	2.2	1.5
Breakfast cereals and cereal bars	1.6	1.6	1.7
Sauces and condiments	1.4	1.3	1.5
Butter, margarine and shortening	1.3	1.3	1.3
Appetizers	1.1	1.1	1.1
Sugar	0.5	0.5	0.6
Other sweets	0.2	0.2	0.3
Jams and other	0.2	0.1	0.2
Sports drinks	0.2	0.2	0.1
Viscera and offal	0.0	0.0	0.0
Other drinks (non-alcoholic)	0.0	0.0	0.0
Unsweetened soft drinks	0.0	0.0	0.0
Coffee and herbal teas	0.0	0.0	0.0
High alcohol content beverages	0.0	0.0	0.0
Energy drinks	0.0	0.0	0.0
Low alcohol content beverages	0.0	0.0	0.0
Supplements and meal replacements	0.0	0.0	0.0
Water	0.0	0.0	0.0

**Table 7:** Dietary sources of energy (%) from food subgroups in adolescents from the ANIBES survey of the Spanish population.

	Adolescents 13–17 Years		
	Total	Men	Women
*n*	211	137	74
Energy (kcal/day)	2018	2124	1823
Bread	11.5	11.7	11.1
Meat	9.2	9.9	7.9
Bakery and pastry	8.4	8.3	8.7
Sausages and other meat products	7.0	7.1	6.7
Olive oil	6.6	6.6	6.6
Ready-to-eat-meals	6.6	6.9	6.0
Milk	5.9	6.2	5.3
Grains and flours	4.7	4.6	4.9
Pasta	4.5	4.9	3.9
Sugared soft drinks	3.4	3.3	3.7
Chocolate	3.3	2.9	4.1
Vegetables	3.0	2.9	3.1
Cheese	2.9	2.7	3.3
Juices and nectars	2.5	2.3	2.9
Fruits	2.4	2.0	3.0
Fish and shellfish	2.1	2.0	2.4
Eggs	2.1	2.2	1.9
Yogurt and fermented milk	2.0	1.8	2.4
Pulses	2.0	1.9	2.1
Breakfast cereals and cereal bars	2.0	2.2	1.6
Other dairy products	2.0	2.1	1.8
Other oils	1.9	1.8	2.2
Sauces and condiments	1.7	1.6	1.7
Butter, margarine and shortening	1.2	1.2	1.3
Appetizers	1.0	0.9	1.2
Sugar	0.7	0.8	0.6
Other sweets	0.2	0.1	0.3
Jams and other	0.1	0.1	0.2
Other drinks (non-alcoholic)	0.1	0.0	0.2
Energy drinks	0.1	0.1	0.0
Low alcohol content beverages	0.0	0.0	0.1
Sports drinks	0.0	0.0	0.1
Coffee and herbal teas	0.0	0.0	0.0
Supplements and meal replacements	0.0	0.0	0.0
Viscera and offal	0.0	0.0	0.0
Unsweetened soft drinks	0.0	0.0	0.0
High alcohol content beverages	0.0	0.0	0.0
Water	0.0	0.0	0.0

**Table 8:** Dietary sources of energy (%) from food subgroups in adults from the ANIBES survey of the Spanish population.

	Adults 18–64 Years		
	Total	Men	Women
*n*	1655	798	857
Energy (kcal/day)	1816	1966	1675
Bread	11.8	12.5	11.2
Meat	9.6	10.2	9.2
Olive oil	9.1	8.6	9.6
Bakery and pastry	6.8	6.3	7.2
Sausages and other meat products	5.9	6.5	5.4
Milk	4.9	4.4	5.3
Fruits	4.7	4.2	5.2
Grains and flours	4.6	4.6	4.7
Ready-to-eat-meals	4.3	4.6	4.1
Vegetables	4.2	4.0	4.4
Pasta	3.7	3.9	3.5
Fish and shellfish	3.7	3.4	3.9
Cheese	3.2	3.1	3.3
Low alcohol content beverages	2.6	3.5	1.8
Yogurt and fermented milk	2.3	2.1	2.5
Pulses	2.3	2.2	2.3
Eggs	2.2	2.4	2.1
Sugared soft drinks	2.1	2.3	1.9
Other oils	1.8	1.8	1.8
Sauces and condiments	1.7	1.7	1.7
Other dairy products	1.5	1.6	1.4
Sugar	1.5	1.3	1.6
Chocolate	1.4	1.3	1.5
Butter, margarine and shortening	1.3	1.1	1.6
Juices and nectars	1.3	1.3	1.2
Breakfast cereals and cereal bars	1.0	0.8	1.1
Appetizers	0.8	0.8	0.8
Jams and other	0.3	0.2	0.4
Other drinks (non-alcoholic)	0.3	0.2	0.4
High alcohol content beverages	0.3	0.3	0.2
Coffee and herbal teas	0.2	0.2	0.2
Viscera and offal	0.1	0.1	0.1
Supplements and meal replacements	0.1	0.1	0.1
Other sweets	0.1	0.1	0.1
Sports drinks	0.1	0.1	0.1
Energy drinks	0.0	0.0	0.0
Unsweetened soft drinks	0.0	0.0	0.0
Water	0.0	0.0	0.0

**Table 9:** Dietary sources of energy (%) from food subgroups in elderly adults from the ANIBES survey of the Spanish population.

	Elderly 65–75 Years		
	Total	Men	Women
*n*	206	99	107
Energy (kcal/day)	1618	1771	1476
Bread	12.5	12.6	12.3
Olive oil	12.2	12.0	12.5
Fruits	8.7	8.1	9.4
Meat	8.5	8.7	8.3
Bakery and pastry	6.4	6.1	6.8
Milk	5.9	5.3	6.4
Vegetables	5.0	4.7	5.2
Fish and shellfish	4.7	4.3	5.0
Sausages and other meat products	4.5	5.1	3.9
Grains and flours	4.1	4.3	3.9
Low alcohol content beverages	3.3	4.8	1.9
Yogurt and fermented milk	3.1	2.5	3.7
Pulses	2.9	3.2	2.5
Eggs	2.8	2.7	2.8
Pasta	2.5	2.1	2.8
Cheese	2.3	1.9	2.6
Butter, margarine and shortening	1.8	1.5	2.2
Ready-to-eat-meals	1.8	2.1	1.6
Sugar	1.4	1.3	1.4
Juices and nectars	0.9	0.9	0.9
Sauces and condiments	0.9	0.7	1.1
Other dairy products	0.8	0.7	1.0
Other oils	0.8	0.8	0.8
Breakfast cereals and cereal bars	0.8	0.7	0.9
Jams and other	0.8	0.6	0.9
Sugared soft drinks	0.7	0.6	0.7
Chocolate	0.5	0.6	0.4
Appetizers	0.3	0.3	0.3
Other drinks (non-alcoholic)	0.3	0.4	0.3
Coffee and herbal teas	0.3	0.2	0.4
High alcohol content beverages	0.3	0.5	0.0
Viscera and offal	0.2	0.2	0.1
Supplements and meal replacements	0.1	0.2	0.0
Sports drinks	0.0	0.0	0.0
Other sweets	0.0	0.0	0.0
Unsweetened soft drinks	0.0	0.0	0.0
Energy drinks	0.0	0.0	0.0
Water	0.0	0.0	0.0

Cereals and cereal products were the main source of energy for the entire sample and all age groups. Within this food group, bread was the major contributor in all age groups (11.6%); this was followed by baked goods and pastries (6.8%), which ranked highest for children and adolescents and much lower for elderly adults. Other minor contributors were grains and flours (4.5%), pasta (3.6%), and breakfast cereals and cereal bars (1.0%). Meat and meat products were the second largest contributor (15.2%), with the lowest ranking for the elderly population (13.1%) and the highest for adolescents (16.2%). Within this category, meat contributed 9.2% of total energy, whereas the sausage and meat derivative subgroup supplied 5.8%. Oils and fats (12.3%) were the third major contributor; these came mainly from olive oil (9.2%) with only 1.7% for other oils and 1.4% for butter, margarine and shortenings. Milk and dairy products contributed 11.8% of total energy intake, and this was higher in children (15.9%) than in adults (11.8%). The different types of milk represented about half the energy intake within this group, followed by cheeses (3.0%), and then closely by yogurt and fermented milk (2.4%). Interestingly, these four food and beverage groups contributed roughly two-thirds (66.7%) of the total energy intake. Much lower contributors included fruits (4.7%), except for in the elderly population (8.7%); ready-to eat meals (4.2%), ranking from 6.6% in adolescents to 1.8% in elderly adults; vegetables (4.0%: 5.0% in elderly adults and 3.0% in children); and non-alcoholic beverages (3.9%: 4.9% in children, 6.1% in adolescents, 3.9% in adults, and 2.2% in elderly adults). Within this category, sugared soft drinks contributed 2% of total daily energy intake (1.9% in children, 3.4% in adolescents, 2.1% in adults, and 0.7% in elderly adults), followed by juices and nectars (1.3%). Fish and shellfish contributed 3.6% of total daily energy intake (2.2%–4.7%, increasing with age group). Sugars and sweets contributed 3.3% for the entire population, and ranked from 4.2% in children to 2.6% in the elderly population. Alcoholic beverages contributed 2.6%, being highest in elderly adults (3.5%). Finally, pulses (2.2%) and eggs (2.2%) had minor contributions to energy intake.

## 4. Discussion

### 4.1. Energy Intake and Profile

There is consensus in the literature that society as a whole is currently in a nutritional transition and there is a need for accurate and updated dietary intake data. Total mean daily energy intake in the ANIBES study is lower than in other surveys like the ENIDE study (“Encuesta Nacional de Ingesta Dietética Española”) [20], a nationwide survey carried out in 2011 with people aged 18–64 where leftovers were not considered as in the ANIBES study. The Food Consumption Survey (FCS), conducted in Spain since 1987, revealed that mean energy consumption for the Spanish adult population in 2010 was 2609 kcal/person/day, which was clearly lower than in 1964 (3008 kcal/person/day) [19,25,26]. However, it should be considered that overestimation may exist in this survey since discards were not recorded. Therefore, our present findings confirm a decreasing trend in energy intake, which has been observed in Spain from different surveys [19,26,27,28] and is consistent with a similar pattern that is occurring in most European countries [29,30,31,32]. When compared with EFSA (European Food Safety Authority 2013) dietary reference values for energy [33] and current (2013) Spanish dietary recommendations for energy [23], intake in the ANIBES study population was only adequate for boys and girls, whereas it was below the average requirement (AR) considering a physical activity level of 1.6 (moderate), for adolescent males (80% of the AR), adult males (78.0% of the AR), and elderly males (77.9% of the AR). In adolescent men, P75 and higher was necessary to guarantee the established AR, and similar was observed for adolescent women. For females, these were 82.6% of the established AR for adolescent women, 82.0% in the case of adult women, and 80.5% for elderly women. It should be considered of special concern that P50 of elderly women in the ANIBES study consumed only 1,426 kcal/day, which may compromise an adequate nutrient-dense diet during the ageing process. In addition, the nutritional status of elderly men (65 to 75 years) may be compromised since only those above P75 reached the adequate AR for energy intake. When national current (2013) dietary recommendations for energy [23] were used for comparison, the results were even more marked in terms of potential insufficient energy intake, with boys only able to cover 81.9% of the recommended dietary intake (RDI); this was 82.3% in the case of girls. Of special note are adolescent men (75.9% of RDI) and women (76.0% of RDI), and particularly adults (69.0% of RDI for men; 79.5% for women) and the elderly population (73.8% of RDI for men; 78.7% for women). It should be considered, however, that these RDI may be insufficiently up to date with respect to stratification of current physical activity levels for the Spanish population.

One of the main dietary quality indices is the energy/caloric profile. In the ANIBES study, protein intake was 16.8%E, well above the upper recommended limit (<15%E). The ENIDE study showed a similar percentage of energy from protein, 18%E, and trends in the Spain FCS are similar [19,20]. Protein intake as a percent of total energy intake ranged from 11.1%E to 17.6%E in the different European countries included in the European Nutrition Health Report (2009) [14]. Fat intake for the total ANIBES study population was 38.5%E, being significantly higher for women. However, there were no age differences in terms of fat contribution to energy, which ranged from 37.2%E in elderly adults to 38.9%E in children. Fat is an important dense source of energy and facilitates the absorption of fat-soluble dietary components, such as vitamins. Fats and oils are also important sources of essential fatty acids. However, high-fat diets may decrease insulin sensitivity and are positively associated with increased cardiovascular risk [34,35,36], although a precise dose-response relationship has not been defined. There is evidence that moderate fat intake (<35%E) is accompanied by reduced energy intake and therefore, moderate weight reduction and/or prevention of weight gain may be better achieved. However, EFSA has concluded that there are insufficient data to define a lower threshold intake (LTI) or tolerable upper intake level (UL) for total fat [37]. Presently, at a European level, a lower boundary for the reference intake range of 20%E and an upper boundary of 35%E have been proposed [37]. A similar range has been recently proposed by WHO and FAO [36].

The SFA intake in the ANIBES study was above the recommendations for all age groups and both genders. SFA are synthetized by the body and are not required in the diet; therefore, no dietary reference intakes have been set. However, there is a positive dose-dependent relationship between intake of a mixture of saturated fatty acids and blood low density lipoprotein (LDL) cholesterol concentrations, when compared with carbohydrates [36]. There is also evidence from dietary intervention studies that decreasing the intake of products rich in saturated fatty acids by replacing them with products rich in *n*-6 PUFA (without changing total fat intake) decreased the number of cardiovascular events [38,39,40]. Because the relationship between increased saturated fatty acid intake and increased LDL cholesterol concentrations is continuous, no threshold of saturated fatty acid intake can be defined below which there is no adverse effect; therefore, no UL can be set, as EFSA has recently established [37]. Even so, the WHO/FAO have recommended that a maximum intake of 10%E for saturated fatty acids should be set [36]. This limit for SFA has also been proposed very recently in the FESNAD Consensus Document on Dietary Fats and Oils for the Adult Spanish Population [41]. Interestingly, the American Heart Association (AHA) has recommended a maximum intake of <7%E for SFA to reduce cardiovascular risk [42]. More recently (2013), the dietary guidelines launched jointly by the AHA and American College of Cardiology proposed a lower amount of energy from SFA (5%–6%E) [43], although there is insufficient scientific evidence proving an association between SFA and cardiovascular and/or diabetes risk.

It is agreed that one positive aspect of the dietary patterns in Spain that should be maintained is the relatively high proportion of MUFA, mostly owing to the common use of olive oil in the Spanish diet [44,45]. In our ANIBES population, MUFA contributed 16.8%E; this was slightly higher in the elderly group and lower in children and adolescents. MUFA intake from energy across Europe ranged from 22% in Greece to 11% in non-olive-oil-consuming countries [37]. The 2011 goals of the Spanish Society of Community Nutrition (SENC) [24] recommended that MUFA should contribute >20%E of total energy. In 2010, an EFSA panel [37] proposed not setting any dietary reference value for MUFA based on the following: MUFA are synthesized by the body, have no known specific role in preventing or promoting diet-related diseases, and are therefore not indispensable constituents of the diet. This assumption by EFSA, however, is untenable as MUFA are among the most abundant fatty acids in most tissue cells and contribute to maintaining membrane fluidity and enzymatic activities. Additionally, there is convincing evidence that MUFA lower both total and LDL plasma cholesterol levels, and replacement of PUFA with MUFA decreases the risk of cardiovascular disease (CVD). Indeed, the FAO/WHO have recommended a MUFA intake of about 16%–19% (obtained by the difference in intake between SFA and PUFA) [36]. Moreover, in the PREDIMED intervention study [46], intake of virgin olive oil (high in MUFA content) was associated with a lower risk of CVD events and total mortality. Interestingly, participants who followed the olive oil-rich Mediterranean diet had a mean MUFA intake of 22%E. Therefore, from the PREDIMED study findings, a MUFA intake target of 20%E–25%E (with virgin olive oil as a main source) is desirable. As for PUFA, in view of the different metabolic effects of the various dietary PUFA [45,47], EFSA has proposed not to formulate a dietary reference value for the intake of total PUFA [37]. Other organizations, such as WHO/FAO in 2010 [36] and SENC (2011) [24], have suggested that PUFA should contribute 6%–10% and 5%, respectively, of total energy intake. In the present study, PUFA contributed roughly 6.6%E, with no gender or age differences. In addition, total *n*-3 PUFA intake expressed as the percentage of energy intake was 0.63%E for the ANIBES study population and increased with age. The WHO/FAO [36] have recommended a minimum intake for adults of 250 mg/day for *n*-3 long-chain PUFA and up to 2 g/day to help prevent CVD.

Intervention studies have provided evidence that high fat (>35%E), low carbohydrate (<50%E) diets are associated with adverse short- and long-term effects on body weight, although the data are insufficient to define an LTI for carbohydrates [47,48]. An EFSA panel [47] therefore reached the conclusion that only a reference intake range can be given, 45%E–60%E, where monosaccharides plus disaccharides should be below 10% of the total energy intake. Data from different dietary surveys have shown that average carbohydrate intakes for children and adolescents in European countries varied between 43%E and 58%E, and from 38%E to 56%E in adults, whereas average sugar intakes varied between 16%E and 36%E in children and adults [14,47]. In the present study, a low energy intake of 41.1% from carbohydrates was seen (17.0% from sugars); a trend was observed according to age, with the lowest contribution in elderly males (39.6%E) and the highest in the youngest age groups (44.4%E). Similar results and trends were obtained for the ENIDE dietary survey in Spain [20]. It is known that frequent consumption of sugar-containing foods can increase the risk of dental caries [49]. However, the available data do not allow the setting of an upper limit for intake of (added) sugars on the basis of risk reduction for dental caries. Evidence relating a high intake of sugars (mainly as added sugars), compared with high starch intakes, to weight gain is also inconsistent [50]. In consequence, according to EFSA, the available data are insufficient to set an upper limit for added sugar intake [36]. Moreover, although there is some evidence that high sugar intakes (>20%E) may increase serum triglyceride and cholesterol concentrations and might adversely affect serum glucose and insulin levels, these data are also insufficient to set an upper limit for (added) sugar intake. The latter does not exclude, however, that food-based dietary guidelines and nutrition goals for the population should take into account the potential negative roles under certain conditions [24,51]. A new WHO guideline [52] recommends that adults and children reduce their daily intake of free sugars to less than 10% of their total energy intake. A further reduction to below 5% has been suggested to provide additional health benefits. The percentage of energy from sugars in our study was 17.0%E for the total population, and was significantly higher in females compared with males and more marked in the oldest participants.

Other minor sources of energy from diet were also estimated. Fiber intake contributed 1.4%E of the total energy, which was significantly higher in females than males. Alcohol intake contribution in the adult populations was considered moderate at 1.9%E. However, alcohol intake is one of the dietary components for which underreporting may occur, especially in women and participants with higher education levels and socioeconomic status [53,54]. In fact, energy contribution from alcohol in men was almost two-fold compared with women. On the other hand, the highest contribution from alcohol corresponded to elderly males (4.1%E), and it was much higher than for elderly women (1.4%E).

### 4.2. Food Sources of Energy

We were able to make the most detailed evaluation to date of how the different food groups and subgroups contribute to energy intake in the Spanish diet. The food group contributing the most to energy consumption was cereals and derivatives (27.4%), regardless of age group or gender. This pattern should be considered positive, but is insufficient for adequate nutrient density (*i.e.*, carbohydrates and dietary fiber intake). Individually, bread was the main contributor, although more efforts are needed to return consumption to levels seen in previous generations of Spaniards, according to the traditional Mediterranean diet [55,56]. Moreover, the baked goods and pastry subgroup closely followed bread, with potentially higher contributions of sugar and unhealthy fats, which was the case for all age groups and especially the youngest (9.4%E). Interestingly, ready-to-eat breakfast cereals and cereal bars contributed most in adolescents, with much lower contribution in the elderly population. Our results also revealed that the contribution of meat and meat products seemed very high for all age groups, which made it difficult to reach the recommended energy and lipid profiles. For comparison, the meats and derivatives group accounted for a total 179 g/adult/day in the last FCS (2012), and has remained steadily high over the last 12 years. Moreover, it should be noted that this food group has increased by roughly 300% when compared with 1960s results in Spain. In addition, caution should be advised since the subgroup that includes sausages and other meat products represented approximately 7.0%E of the total energy in children and adolescents from the ANIBES sample, which was quite different from the elderly group (4.5%E).

The next main group contributing to energy intake was oils and fats (12.3%E), but age-marked differences were seen. The lowest contribution was in children and adolescents accounting for 10%E, but this was 15%E in elderly adults; this means that a “missing” percentage of the energy from oils and fats in the youngest age groups may be replaced by meat and meat products. Fortunately, olive oil represented the main contributor, with nearly 10%E of total intake. Milk and dairy products were next in energy contribution, showing a clear decreasing trend with advancing age. As expected, milk was the main subgroup, although a decreasing trend in milk consumption has been observed in Spain in recent years. In fact, a significant decrease in the amount of purchased dairy products has taken place from the years 2000 (416 g/person/day) to 2012 (359 g/person/day), according to the FCS [19,26]. One of the main concerns is the possibility that milk is being replaced by other less nutrient-dense foods and beverages, mainly in younger age groups. In the ENIDE dietary survey during 2011 in Spain [14], the main sources of energy were meat and meat products (18%), followed by cereals and derivatives (17%), oils and fats (12%), and milk and dairy products (11%). According to the Spain FCS, the food groups contributing most to energy consumption were cereals and derivatives (24.6%), meat and meat products (14.3%), oils and fats (13.6%) and milk and derivatives (12.5%). By contrast, fish and shellfish (3%), non-alcoholic beverages (2.9%), and alcoholic beverages (2.3%) showed a lower contribution to total energy intake. By comparison, data from other European countries (e.g., Nordic countries) showed that cereals and milk and dairy products are usually the main energy sources, ahead of the meat and derivatives group [57,58].

In the present ANIBES study, the following contributing groups showed a marked gap, since the sum of the remaining disaggregated 35 food and beverage groups and subgroups was only 33.2% (600 kcal/day) of the total energy intake. Interestingly, the contribution of fish to total energy intake was only 3.6% and increased markedly with advancing age. This may compensate for younger age groups, to achieve current dietary guidelines for fish consumption [24]. The FCS showed similar results for fish and shellfish as sources of energy (3.0%). However, the ENIDE results were much higher (9%) for fish, shellfish, and derivatives in the adult population [14].

The impact of sugared soft drink consumption on obesity and metabolic disorders has come under intense scrutiny and debate worldwide in recent years [59,60,61,62], and large differences between countries have been observed. The present study showed that sugared soft drinks contributed 2.0% (36 kcal/day out of 1810 kcal/day) to total energy intake. A lower consumption compared with mean contribution was seen in children (1.9%E, 34 kcal/day) whereas the lowest contribution was for the elderly population (0.7%E, 13 kcal/day). Higher consumptions were found, however, for adolescents (3.4%E, 61 kcal/day) and the contribution in adults was 2.1%E, 38 kcal/day. Using FCS data, we have previously shown [17] that all non-alcoholic drinks contributed 2.9% to total energy intake in Spain. For additional comparison, in the ENIDE dietary survey, non-dairy beverages (excluding alcoholic drinks) contributed 2%E (46 kcal/day) in the adult Spanish population [20]. By contrast, the United States has usually had the highest contribution to energy intake from sweetened beverages. However, a recent study using data from NHANES surveys showed that from 1988 to 1994 and 1999 to 2004, the consumption of these beverages increased [63], but consumption of beverages and foods with added sugars declined from 1999 to 2000 [64]. Another food group of concern, which is usually not well quantified, is that of so-called sugars and sweets, mainly owing to its potential role in overweight, obesity, and several metabolic disorders [59,60,61,62]. We showed that this group currently represents 3.3% of total energy intake in Spain and decreases with age (5.1% in children and 2.6% in elderly adults). In the ENIDE survey, this food group contributed 5% to total energy intake [20].

Finally, we found lower contribution than initially expected from alcoholic drinks (2.6%E, 47 kcal/day), which was higher for elderly participants. Alcoholic beverages of lower alcohol content (beer, wine, cider) represented over 90% of energy contribution within this group. These results are similar to those obtained from the latest Spanish FCS (2.3%E) [19]. In general, alcoholic beverage consumption has undergone a slow decline during recent years (259 g/person/day in 2000 *versus* 208 g/person/day in 2012) [19]. Within this group, as a beverage traditionally included in the Mediterranean diet concept, wine only represented 23.5% of total alcoholic beverage consumption whereas it accounted for 62% of the total consumption in 1991. In the last few years, a gradual substitution of wine for beer has taken place, which represents almost 70% of the total alcoholic beverage consumption at present [19].

## 5. Conclusions

To summarize, the top 10 food groups and subgroups of energy sources in the Spanish diet were (in decreasing order): bread, olive oil, fresh meat, baked goods and pastry, sausages and other processed meats, milks, fruits, ready-to-eat meals, vegetables, and grains and flours. These accounted for about two-thirds of total energy intake, whereas the remaining 33% of the energy from foods and beverages was widely distributed among 35 different food and beverage subgroups. In conclusion, the strengths of the design, protocol and methodology used in the ANIBES study are the representative national sample targeted, the broad age range included (9–75 years), the geographical distribution (mainland and islands), successful logistics for the 128 sampling points, and innovative and novel use of tools to measure dietary intake and leftovers. However, the ANIBES study had some limitations, mainly difficulties for some participants (e.g., elderly adults) in using new technology to record intakes. There was also no accounting for seasonality in food consumption. Finally, although ANIBES data were representative of the Spanish population, caution should be used in inferring causal relationships between diet quality, body weight, and other health outcomes. Despite the limitations, these data are the best available to evaluate current dietary energy intake and its determinants for the Spanish population.
